# Study on the Influence of Au Content and Bonding Parameters on the Free Air Ball Morphology and Bonding Reliability of Ag-Au-Pd Alloy Wire

**DOI:** 10.3390/mi15121512

**Published:** 2024-12-20

**Authors:** Junling Fan, Fang He, Bing Chen, Junchao Zhang, Fan Yang, Jun Cao, Furong Wang

**Affiliations:** 1 School of Chemical and Environmental Engineering, Jiaozuo University, Jiaozuo 454000, China; hefang1182@163.com (F.H.); cohobo@126.com (B.C.); 2 School of Mechanical and Power Engineering, Henan Polytechnic University, Jiaozuo 454000, China; zjc_xlyx@sina.com (J.Z.); yangfan2442@163.com (F.Y.); cavan@hpu.edu.cn (J.C.); wangfr@hpu.edu.cn (F.W.)

**Keywords:** bonding parameters, FAB characteristics, failure probability

## Abstract

This article conducts wire bonding tests and cold/hot-cycle tests using φ 0.025 mm Ag-Au alloy wires and Ag-Au-Pd alloy wires with different specifications. The results show that, due to the addition of the alloying element Pd, under the same bonding parameters, the fracture strength and ball-bonded point shear force of the Ag-Au-Pd alloy wires are significantly higher than those of the Ag-Au alloy wires. After the cold/hot-cycle tests, the failure probability of the Ag-Au-Pd alloy wires is approximately half that of the Ag-Au alloy wires. Among Ag-Au-Pd alloy wires, 92% break at the ideal positions, while 77% of the Ag-Au alloy wires break at the necks. As the Au content increases, the Free Air Ball (FAB) morphology of the Ag-Au-Pd alloy wires becomes more and more regular, gradually transitioning from a pointed ball to an ellipsoid and finally presenting a spherical shape.

## 1. Introduction

Bonding wires are essential and crucial materials for integrated circuit (IC) packaging, and they play roles in both signal transmission and heat transfer and dissipation. Ultra-fine-pitch wire bonding requires wires with a finer diameter and higher strength and reliability. Through the control of the Free Air Ball (FAB) and the length of the heat-affected-zone, connection strength is generated under the combined action of ultrasonic energy, pressure, and high temperature during the bonding process to meet the needs of chip interconnection [1,2,3]. Bonding Ag wires have excellent electrical properties (such as reducing the high-frequency noise of devices and heat generation of high-power devices), an appropriate cost. They can effectively reduce light attenuation and improve conversion rate in LED packaging. These numerous advantages make them start to be applied in microelectronic packaging [4,5,6]. However, Ag wires also have the following problems: (1) They have relatively low strength, and are prone to defects such as wire collapse and unstable wire arc in low-arc-height wire bonding packaging, and cannot meet the requirements for the packaging of low-arc-height and high-density devices [7]; (2) they have low strength under high-temperature conditions and a relatively high failure probability, and cannot meet the usage requirements of devices such as high-power LEDs [8]; (3) Under high-temperature and high-humidity conditions, the Ag/Al bonding interface is prone to Ag+ electromigration, which leads to a decrease in the strength of the bonding points and thus affects the lifespan of devices [9,10,11,12]. Additionally, the long-term use of pure Ag wire is prone to sulfide and chlorination phenomena, resulting in low bonding reliability [13].

Obtaining high-performance Ag-based bonding alloy wires through alloying is an effective way to improve the properties of bonding Ag wires. Elements Au and Pd have similar characteristics to Ag and are infinitely miscible. The addition of Au and Pd elements can increase the strength and high-temperature stability of Ag bonding wires [14], expand the parameter window range and interface connection strength during the bonding process [15], and have an inhibitory effect on the growth of intermetallic compounds at the bonding interface (especially under high-temperature and high-humidity conditions), which is conducive to further enhancing the reliability of the bonding interface and prolonging the lifespan of devices [16,17]. The study by Yuan et al. [18] also confirms that the addition of Pd can improve the mechanical properties of Ag wire. Kuo et al. [19] found that the hardness and tensile strength of Ag-based alloy wires increase with the increase in Au content. Cho et al. [20] found via experimental studies that interfacial corrosion was significantly inhibited with an increase in Pd content, and the reliability of Ag wire bonding containing Pd was significantly improved.

Ag-Au-Pd bonding alloy wire compensates for the insufficient comprehensive performance of single element bonding wires such as bonding alloy wire, copper wire, silver wire, etc., in the application process [21,22,23]. It holds great potential for application in chip packaging for high-density, large-scale integrated circuits and high-power LEDs. However, for Ag-Au-Pd bonded alloy wires, due to the influence of alloy elements, there are irregular shapes such as pointed balls, off-center balls, and golf clubs during the FAB generation process. Irregular FAB will lead to the insufficient bonding strength of the first solder joint or cause connection defects such as short circuits and virtual soldering, increasing the probability of device failure. This limits the application of Ag–Au–Pd bonded alloy wires in aerospace, automotive, military, and other fields. Therefore, it is necessary to explore in depth the characteristics and influencing factors of FAB generation in Ag-Au-Pd bonded alloy wires, in order to solve the problem of the high probability of FAB defects in Ag-Au-Pd during the bonding process, and improve the application efficiency and device reliability of Ag-Au-Pd bonded alloy wires. In view of this, this article conducted key technical research on the shape–related characteristics and bonding strength of Ag-Au-Pd bonded alloy wire FAB, providing effective technical means for controlling the shape of Ag-Au-Pd bonded alloy wire FAB and breaking through the bottleneck of the difficult defect control of Ag-Au-Pd bonded alloy wire FAB.

## 2. Experimental

**Figure 1:**
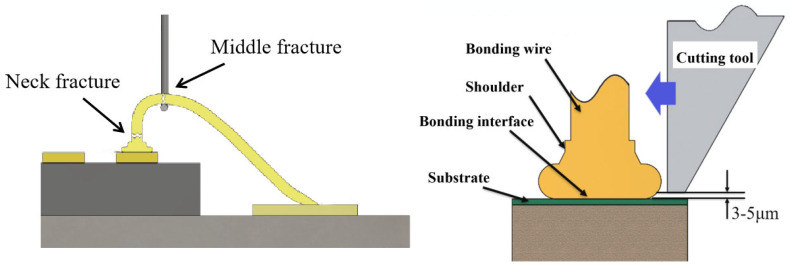
Bonded wire tension force test and ball-bonded point shear force test [25].

The test materials are φ 0.025 mm Ag-Au alloy wires and Ag-Au-Pd alloy wires with four different Au contents. The Au contents in the four types of Ag-Au-Pd alloy wires are as follows: 1#: 1%; 2#: 1.48%; 3#: 2.51%; 4#: 3.84%. Bonding tests and Electronic Flame Off (EFO) tests of the Ag-Au alloy wires and Ag-Au-Pd alloy wires were carried out on an automatic bonding device, and the bonding parameters are shown in Table 1. The bonding parameters of 2#, 3# and 4# Ag-Au-Pd wires with different mechanical properties (strength and elongation) are shown in Table 2. Bonding device model: KAIJO FB-988 (The manufacturer is Iwase Corporation, Gunma Prefecture, Japan); Package chip type: 2835 LED; N_2_ gas protection is adopted during the bonding process with a gas flow rate of 0.6 L/min; The Dage Series 4000, Bs250 tester is used to conduct bonded wire tensile and ball-bonded point shear force tests on the bonded samples, and the testing method referred to the relevant literature [24]. The test data are statistically analyzed, as shown in the Figure 1. The bonded samples were packaged with resin, and subjected to cold/hot cycle tests through an ESPEC TSE-11 cold/hot-cycle chamber. The sample was preserved at a high temperature of 100 °C for 30 min and at a low temperature of −40 °C for 30 min as one cycle, and cold/hot-cycle tests were conducted for 100, 200, and 300 times, respectively, and then the failure rate was detected [25]. The JEOL JSM-6700F scanning electron microscope (SEM) (The manufacturer is Nippon Electronics Corporation, Tokyo, Japan) was used to observe the state of the samples after decapsulation (working voltage 15–20 kV, working distance 7–12 mm), and the FAB shapes and bonding morphologies of the bonding wires were analyzed with different gold contents.

## 3. Results and Discussion

### 3.1. Study on Wire Bonding Strength of Ag-Au and Ag-Au-Pd Alloys

**Figure 2:**
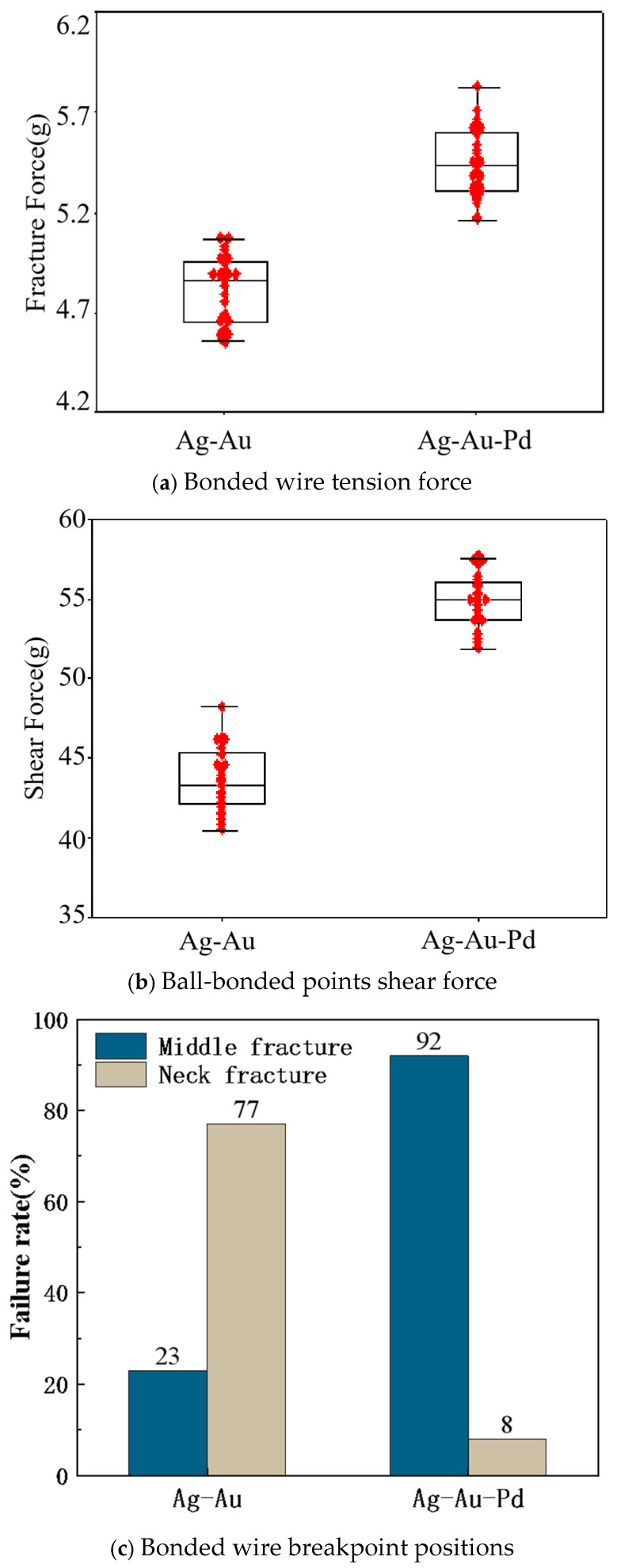
The tension force, shear force and breakpoint positions of Ag-Au and 1# alloy wires.

Figure 2a shows the bonded wire tensile force distribution of Ag-Au and 1# alloy wires under the same bonding parameters. The average tensile forces of Ag-Au and 1# alloy wires are 4.81 g and 5.44 g, respectively. The breaking tensile force of Ag-Au wires is relatively concentrated, but the value is relatively small (all below 5 g). Although the breaking tensile force distribution of 1# alloy wires is relatively dispersed, its minimum value is also above 5 g. Figure 2b shows the ball-bonded point shear force distribution of Ag-Au and 1# alloy wires, and their average shear forces are 43.64 g and 54.83 g, respectively. The ball-bonded point shear force data of Ag-Au wires are relatively dispersed, while the shear force data of 1# alloy wires are concentrated and the value is obviously higher than that of Ag-Au wires. Figure 2c shows the breakpoint position statistics of Ag-Au and 1# alloy wires. For Ag-Au wires, 77% of the bonded wire tensile force breakpoints are at the neck (neck breakage is caused by low neck strength and is a form of device failure), and 23% of the breakpoints are the middle breakages (the ideal breakpoint in bonded wire tensile force tests, indicating that the neck strength at the ball-bonded point is higher than that of the wire itself) [25]. For 1# alloy wires, 92% of the bonded wire tensile-force breakpoints are at the neck, and 8% of the breakpoints are the middle breakages. This proves that the bonding reliability of 1# alloy wires is higher than that of Ag-Au wires.

Ag alloy wires with various contents of Pd and Au were evaluated by Tsai et al. [26]. The results showed that their breaking force increases with Au and Pd contents. Yuan et al. [18] compared Ag alloy wires with different Pd contents and pure Ag wires and found that the addition of Pd improved the mechanical properties of Ag wires. Adding Au to the Ag-Pd alloy wire will improve the thermal stability of the Ag alloy wire. Comparative analysis reveals that the bonded wire tensile-forces and ball-bonded point shear forces of 1# alloy wire balls surpass those of Ag-Au alloy wires. This is mainly due to the fact that, after the addition of Pd elements to the Ag-Au alloy, the substitution relationship between Pd atoms and Ag atoms results in more elastic distortion between atoms. When dislocations move to the distortion area under the action of external force, due to the effect of distortion energy, the movement of dislocations will be hindered. At this time, a greater external force must be applied to overcome this distortion resistance, so that both of the bonded wire tensile force and ball-bonded point shear force value of 1# alloy wire is higher than that of the Ag-Au alloy wire.

In addition, during the bonding process, under the action of ultrasonic energy and pressure, the dislocation density at the bonded point increases dramatically and causes lattice distortion. The bonding interface has a relatively low diffusion activation energy, and atoms diffuse rapidly along the dislocations on the contact surface, forming a certain connection strength. Due to the addition of Pd in Ag-Au-Pd alloy wire, the dislocation density caused by alloy elements at the bonding interface increases, and its atomic diffusion rate at the bonding interface is higher than that of the Ag-Au alloy wire. Therefore, the shear force of the ball-bonded point of the 1# alloy wire is relatively high, and the ball bonded point has a large amount of residual crystals after the shearing. Moreover, for the 1# alloy wire, due to the addition of Pd alloy element, the recrystallization temperature of the 1# alloy wire is higher than that of the Ag-Au alloy wire. The length of the heat-affected-zone at the FAB neck of the 1# alloy wire is shorter than that of the Ag-Au alloy wire, so the probability of fracture at the neck during the bonded wire tension force test is relatively small. However, for the Ag-Au wire, due to its relatively long heat-affected-zone length, the neck strength is severely reduced, and thus the probability of neck fracture is relatively large.

### 3.2. Bonding Reliability Study of Ag-Au and 1# Alloy Wire

**Figure 3:**
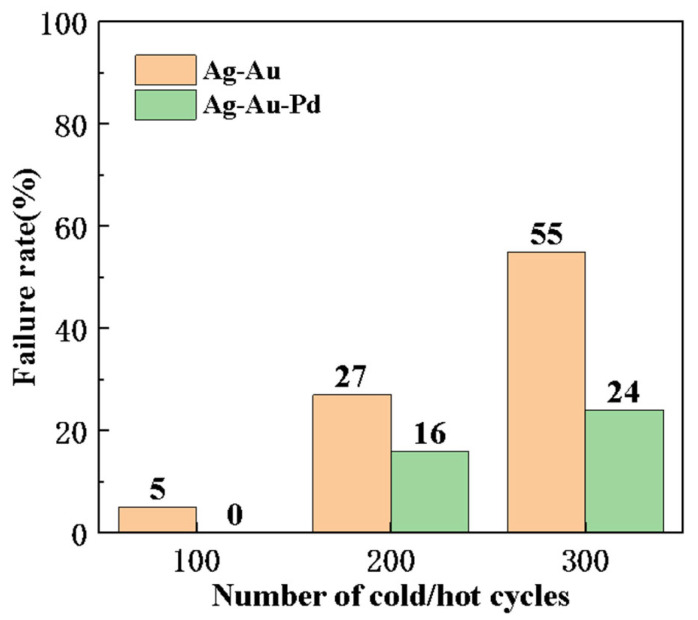
Failure ratio of Ag-Au and 1# alloy wires.

Figure 3 shows the failure ratios of Ag-Au and 1# alloy wire boned samples after the cold/hot-cycle test. After 100 cold/hot-cycle tests, 5% of the Ag-Au alloy wires failed, while the 1# alloy wire did not show any failure. After 200 cold/hot-cycle tests, 27% of the Ag-Au alloy wires failed, and the 1# alloy wires also had a failure rate of 16%. After 300 cold/hot-cycle tests, more than half (55%) of the Ag-Au alloy wires failed; while only a quarter of the 1# alloy wires failed, and the failure rate was less than half of that of the Ag-Au alloy wires.

**Figure 4:**
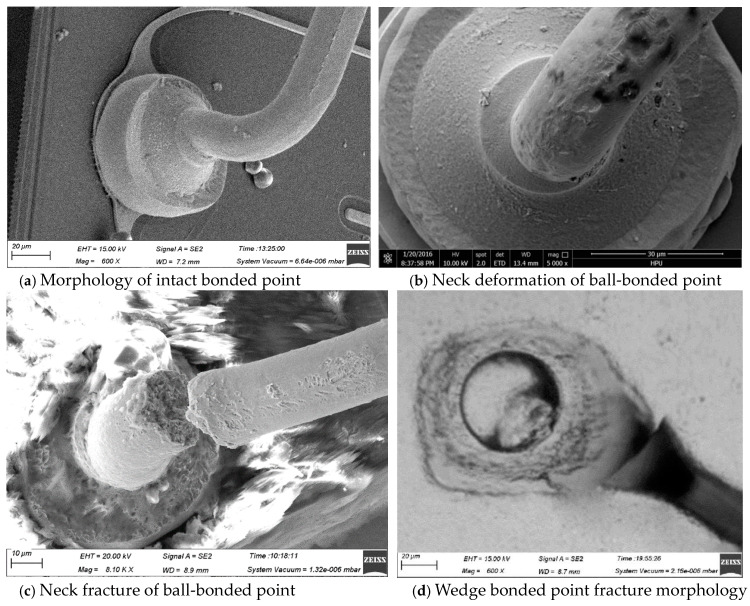
Morphology of Ag-Au alloy wires bonded points.

**Figure 5:**
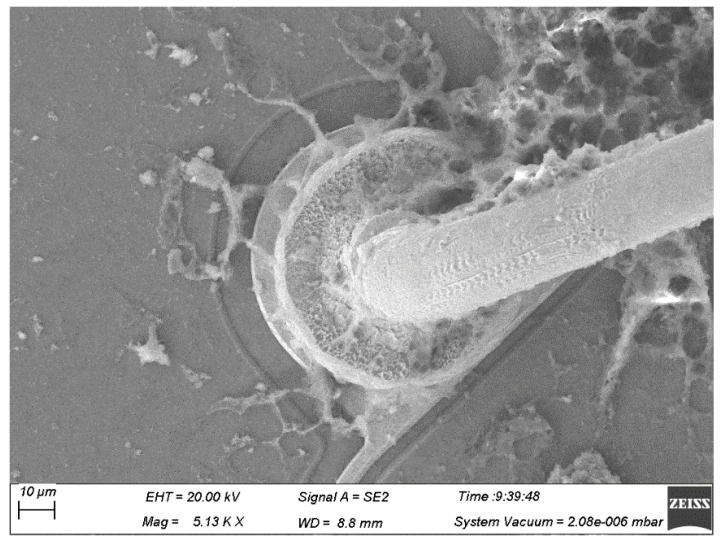
Morphology of the intact ball-bonded point of 1# alloy wire.

Figure 4 illustrates the intact bonded point, neck twisting, and fracture failure of Ag-Au alloy wire ball-bonded points that were unsealed after undergoing cold/hot-cycle tests, respectively. As can be seen from Figure 4b,c, the failure of the Ag-Au alloy wire is manifested as the neck twisting and the fracturing of the ball-bonded point. During the bonding process, the neck of the ball-bonded point needs to be repeatedly deformed greatly to complete the wire-bonded-arc formation. For the bonded Ag-Au alloy wire, due to the long length of the neck heat-affected zone and the coarse internal grains, its plasticity is poor. When the neck is rapidly bent, a large deformation will occur, resulting in severe stress concentration accompanied by slip, which is likely to cause the generation of micro-cracks. In the subsequent cold/hot-cycle tests, the micro-cracks are continuously strengthened, resulting in neck fracture, as shown in Figure 4c. In addition, for the bonded Ag-Au alloy wire, because of its relatively weak strength, it is more likely to be twisted at the neck, thereby increasing the probability of neck fracture, as shown in Figure 4b. Moreover, during the wedge-bonding process, due to the action of the bonding pressure, the amount of deformation at its neck is relatively large, and the neck of the wedge bonding point fractures after the cold/hot-cycles process, as shown in Figure 4d. For the 1# alloy wire, due to the shorter length of the neck heat-affected-zone and better plastic deformation capability, it retains good mechanical properties, even after multiple cold/hot-cycles, resulting in a lower likelihood of fracture, as shown in Figure 5.

### 3.3. Study on the Influence of EFO Parameters on the FAB Shape of Ag-Au-Pd Alloy Wires with Different Au Contents

**Figure 6:**
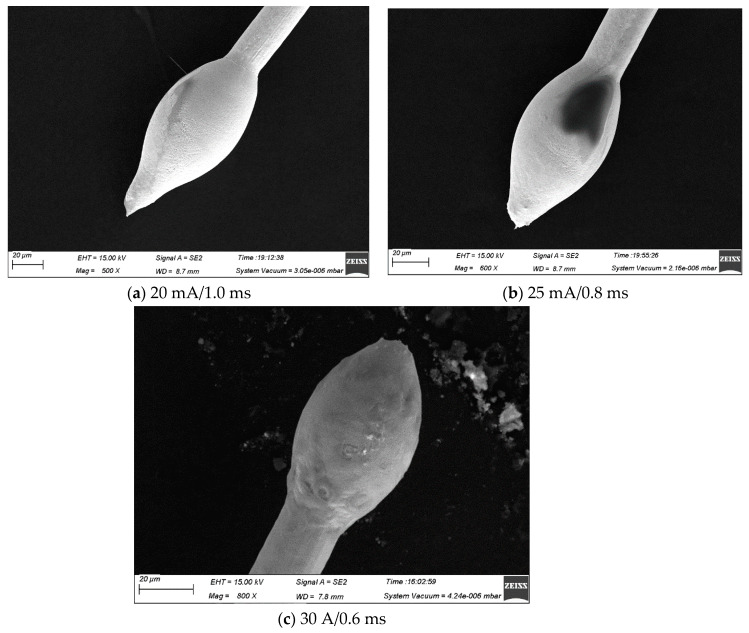
FAB morphology of 2# alloy wire under different bonding parameters ((**a**) small conical shape, (**b**) small conical shape, (**c**) ellipsoidal shape).

**Figure 7:**
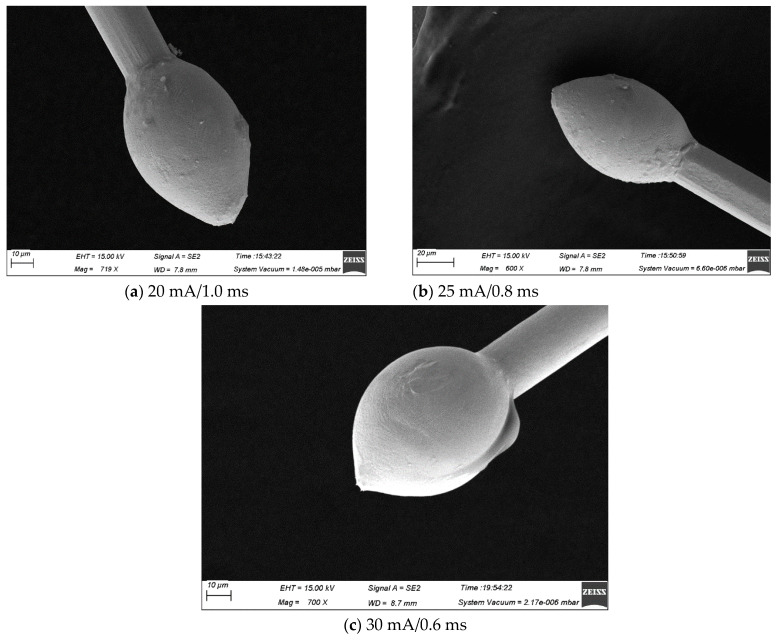
FAB morphology of 3 # alloy wire under different bonding parameters ((**a**) small ellipsoid, (**b**) small ellipsoid, (**c**) large ellipsoid).

**Figure 8:**
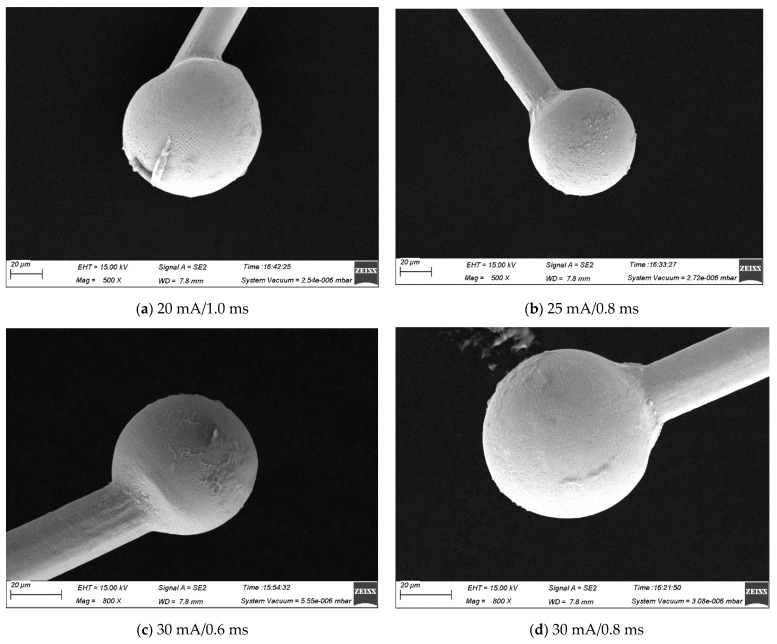
FAB morphology of 4# alloy wire under different bonding parameters ((**a**) golf club shaped, (**b**) small conical spherical, and (**c**) large elliptical); (**d**) big round spherical.

Over the years, several studies have investigated the ball-forming properties of wire bonds [27,28]. They found that EFO current and time are the two most critical parameters in the balling process. Figure 6, Figure 7 and Figure 8 show the FAB morphologies of alloy wires 2#, 3#, and 4# under different EFO currents and times. As can be seen from Figure 6a, the FAB of 2# alloy wire is generally in the shape of a sharp cone. After increasing the EFO current and shortening the EFO time, there is almost no significant change in the FAB, as shown in Figure 6b. Since the obtained energy only melts a small amount at the wire tail, the FAB cannot become spherical. By increasing the EFO current again and shortening the EFO time, the FAB presents a small ellipsoid shape, but it is still not a normal spherical shape, as shown in Figure 6c. The FAB of 3# alloy wire is a small ellipsoid shape, as shown in Figure 7a,b. After increasing the EFO current and shortening the time, the FAB becomes more plump and the size also increases slightly, but it still presents an ellipsoid shape, as shown in Figure 7c. Under the EFO parameters of the small current and long time, the FAB of the 4# alloy wire is in the shape of a golf club, as shown in Figure 8a. After increasing the EFO current and shortening the EFO time, the spherical shape and size are qualified, as shown in Figure 8b,c, which can all meet the bonding requirements. It can be seen that, as the current and time of EFO increase, more heat was generated, which allowed more of the wire to be melted, resulting in a larger average diameter for the FAB [29,30].

However, under a relatively large current and a long EFO time, the FAB of 4# alloy wire still shows a regular spherical shape, with a slightly larger size but still within a reasonable range, as shown in Figure 8d. Therefore, the increase in Au content indeed reduces the difficulty of FAB formation for alloy wires. The Ag-Au-Pd wire with a high Au content has a wide range of EFO parameters, a regular FAB morphology, and higher tissue uniformity.

### 3.4. Study on the Influence of 4# Alloy Wire with Different Mechanical Properties on Ball Bond Point Strength

**Figure 9:**
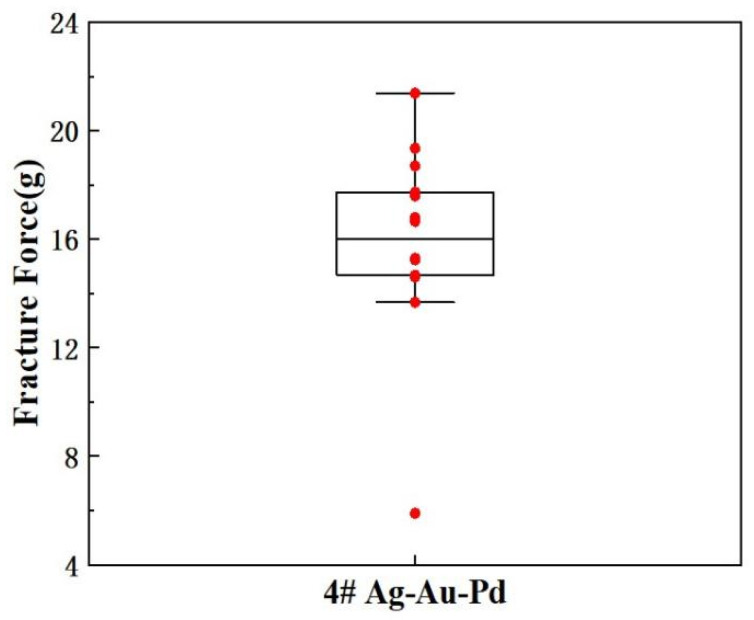
Bonded wire tensile force statistics of 4 # Ag-Au Pd wire.

Figure 9 shows the statistical analysis results of the bonded wire tensile force test using annealed soft-state 4# alloy wire. The range of the tensile force values of the 4# alloy wire fluctuates greatly, with the minimum value being 5.89 g and the maximum value being 21.38 g, but the minimum value is relatively low.

**Figure 10:**
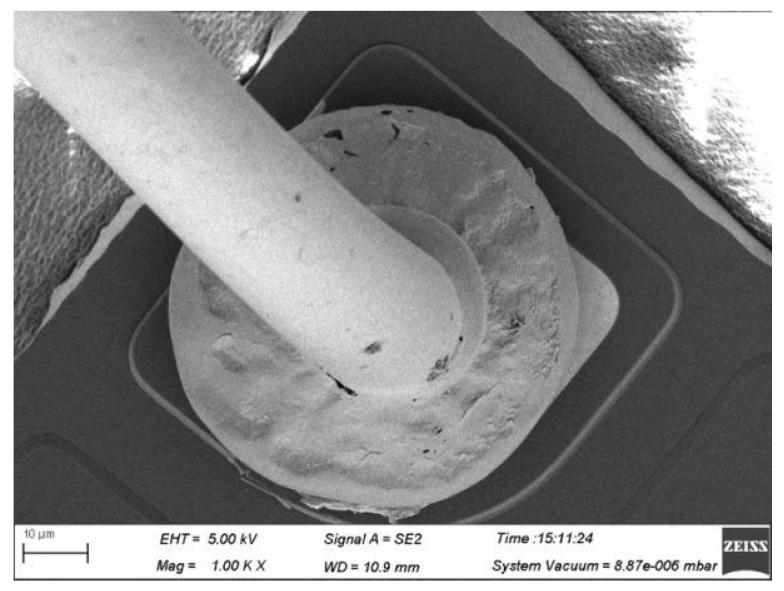
Morphology of ball-bonded points of 4# alloy wire.

**Figure 11:**
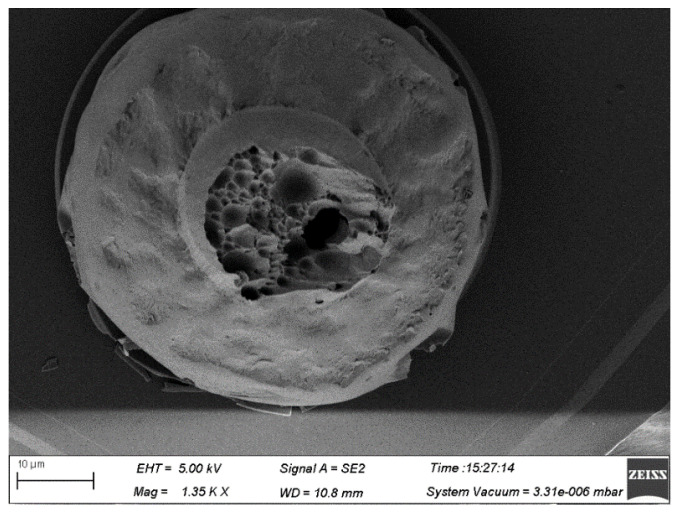
Breakpoint morphology of 4# alloy wire ball bonded point.

**Figure 12:**
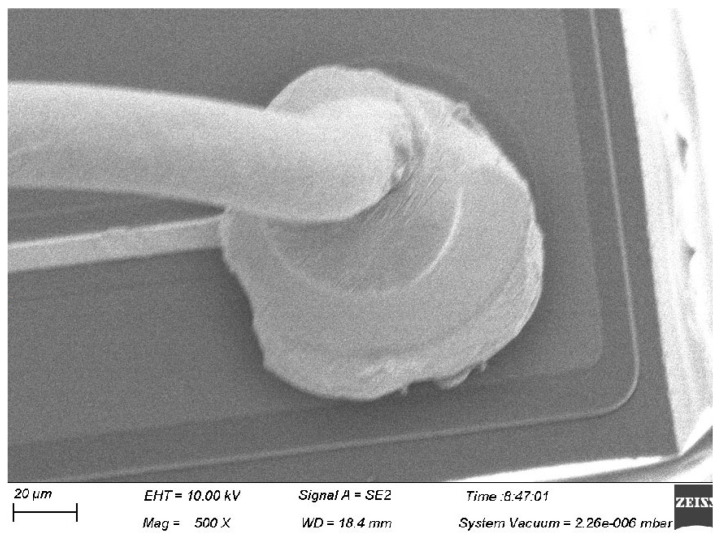
Morphology of wire ball-bonded points in hard Ag-Au alloy (with cracks at the neck).

For the soft state 4# alloy wire, the bonded wire tensile force is unstable after wire bonding. According to the analysis in Figure 10 and Figure 11, the grains in the neck of this alloy wire are relatively large, resulting in a relatively low strength in the neck of the ball-bonded point. As for the hard-state 4# alloy wire, due to its low elongation rate, there is a large stress concentration in the neck during the bonding process, thus leading to neck cracks, as shown in Figure 12. From the above results, it can be seen that the failure rate of hard state 1# alloy wires is relatively high during the bonding process. Adjusting the heat treatment parameters appropriately can improve the performance of Ag-Au-Pd alloy wires and enhance the bonding effect.

## 4. Conclusions

Through the research on Ag-Au-Pd alloy wires, the following conclusions are drawn:

(1) Under the same bonding parameters, both the bonded wire tensile force and ball-bonded point shear force of 1# alloy wire are greater than those of the Ag-Au alloy wire. The heat-affected-zone at the neck of the 1# alloy wire ball-bonded point is shorter, and the failure rate of its samples is lower.
(2) After 300 times cold/hot-cycle tests, the failure probability of 1# alloy wire is approximately 24%, less than half of that of Ag-Au alloy wire. Moreover, 92% of the fracture positions of 1# alloy wire are in the middle, while 77% of the fracture positions of Ag-Au alloy wire are at the neck.
(3) By appropriately increasing the gold content and adjusting the heat treatment parameters, the FAB morphology and bonding performance of Ag-Au-Pd alloy wires can be effectively improved. When the Au content is 3.84%, the FAB shape of 1# alloy wire is spherical.

## Figures and Tables

**Table 1:** Bonding parameters of Ag-Au wire.

Ball Bonding		Wedge Bonding		EFO	
Bonding pressure	0.45 N	Bonding pressure	0.75 N	Voltage	5000 V
Ultrasonic power	65 mW	Ultrasonic power	95 mW	Current	300 mA
Ultrasonic time	12 ms	Ultrasonic time	12 ms	Time	0.75 ms
		Bonding pressure	0.65 N		
		Ultrasonic power	95 mW		
		Ultrasonic time	12 ms		

**Table 2:** Bonding Parameters of Ag-Au-Pd Alloy Wire.

Ball Bonding		Wedge Bonding		EFO	
Bonding pressure	0.70 N	Bonding pressure	0.85 N	Voltage	5000 V
Ultrasonic power	55 mW	Ultrasonic power	70 mW	Current	20/25/30 mA
Ultrasonic time	8 ms	Ultrasonic time	6 ms	Time	1.0/0.8/0.6 ms
		Bonding pressure	0.70 N		
		Ultrasonic power	50 mW		
		Ultrasonic time	6 ms		

## Data Availability

The data used to support the findings of this study are included within the article.
